# The promise of immunonutrition in pediatric pulmonary rehabilitation

**DOI:** 10.3389/fnut.2026.1769202

**Published:** 2026-05-21

**Authors:** Liang Pan, Ailin Wang, Yanshan Zhang, Xiang Jia, Sixun Yu, Dongyang Liu

**Affiliations:** 1 Department of Pediatrics, Western Theater General Hospital, Chengdu, Sichuan, China; 2 Department of Neurosurgery, Western Theater General Hospital, Chengdu, Sichuan, China

**Keywords:** Gut-Lung Axis, Immunonutrition, Omega-3 fatty Acids, Pediatric Pulmonary Rehabilitation, Vitamin D

## Abstract

The management of pediatric pulmonary diseases is increasingly evolving from conventional respiratory and physical interventions toward integrated strategies that incorporate nutritional and immunomodulatory approaches. Childhood represents a critical window for immune system development and maturation, during which nutritional status profoundly influences immune responses, inflammatory processes, and tissue repair—largely mediated through the gut–lung axis and multiple molecular pathways. This review comprehensively reviews the immunomodulatory roles of key nutrients—including vitamin D, ω-3 polyunsaturated fatty acids, prebiotics/probiotics, and branched-chain amino acids—in supporting lung rehabilitation in children. It elaborates on how these nutrients modulate innate and adaptive immunity, influence key inflammatory markers such as TNF-α and IL-6, and optimize the microecological milieu, thereby facilitating pulmonary function recovery, enhancing exercise tolerance and respiratory muscle strength, and reducing the risk of recurrent infections. Although compelling mechanistic insights have been provided by preclinical studies and some adult clinical trials, high-quality randomized controlled trials in pediatric populations remain limited. Moreover, existing evidence exhibits considerable heterogeneity in terms of intervention dosage, formulation, and individual responsiveness. Future research should prioritize well-designed, multicenter trials on precision nutrition interventions for children, incorporating advanced tools such as nanotechnology, metabolomics, and digital health technologies to establish standardized and personalized immunonutritional frameworks for pediatric pulmonary rehabilitation.

## 1 Introduction

Pediatric pulmonary diseases, such as asthma, bronchopulmonary dysplasia, cystic fibrosis, and recurrent respiratory tract infections, represent prevalent conditions that significantly impact child health and development. Conventional rehabilitation strategies have primarily centered on respiratory therapy, pharmacological interventions, and physical training. While these approaches yield certain benefits, a considerable number of children continue to experience suboptimal recovery of lung function, diminished quality of life, and unfavorable long-term outcomes

Recent advances in immunology and nutritional science have underscored the pivotal role of nutritional status in modulating immune function, thereby critically influencing disease progression and rehabilitation efficacy. Childhood constitutes a crucial “window of opportunity” for immune maturation and metabolic programming, during which the growing organism is particularly vulnerable to nutrient deficiencies or imbalances. A body of evidence indicates that targeted nutritional interventions—including proteins, amino acids, fatty acids, vitamins, minerals, and prebiotics/probiotics—can enhance both innate and adaptive immune responses through multiple pathways, ultimately shaping inflammatory processes and tissue repair (1–3).

On one hand, inadequate or unbalanced nutrition may disrupt immunometabolic homeostasis, impair the function of macrophages, dendritic cells, and T lymphocytes, exacerbate inflammatory responses, and delay the repair of lung tissue. On the other hand, appropriate nutritional supplementation has been shown to modulate inflammatory mediators, promote the secretion of anti-inflammatory cytokines, reinforce barrier integrity, and optimize the microecological milieu—collectively supporting pulmonary recovery and reducing the risk of reinfection (4–6). Against this backdrop, the concept of “immunonutrition” has emerged, advocating the strategic use of specific immunomodulatory nutrients to precisely regulate inflammatory cascades, bolster anti-pathogen defense, and facilitate tissue healing (7).

**Figure 1:**
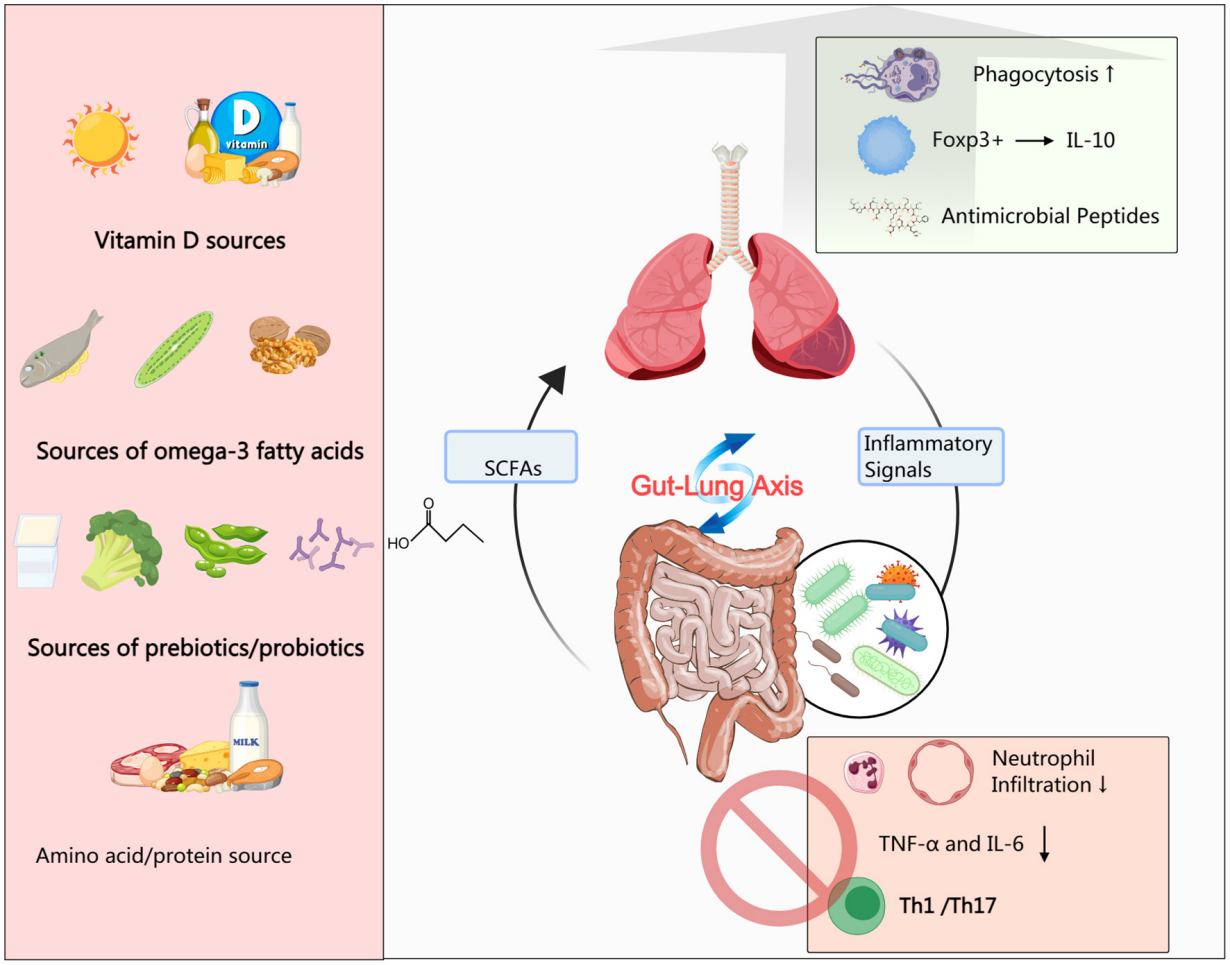
Mechanisms of immunonutrition in pediatric pulmonary rehabilitation. Key nutrients (Vitamin D, Omega-3 PUFAs, Pre/Probiotics, and Amino Acids/Proteins) mediate their effects through the gut-lung axis and systemic immunomodulation. Short-chain fatty acids (SCFAs) and other microbial metabolites from the gut influence lung immunity, while nutrients directly promote anti-inflammatory and pro-resolving processes (e.g., Treg differentiation, SPM production) and inhibit pro-inflammatory pathways (e.g., neutrophil infiltration, Th1/Th17 responses). These coordinated actions contribute to improved lung function, reduced reinfection risk, and enhanced overall recovery. Created with MedPeer (medpeer.cn).

This review aims to comprehensively elucidate the mechanisms and clinical evidence supporting the use of key nutrients—vitamin D, ω-3 polyunsaturated fatty acids, prebiotics/probiotics, and select amino acids—in the context of immunomodulation during pediatric pulmonary rehabilitation. We will focus on the crosstalk between microbial ecology and host immunity mediated via the gut–lung axis, examine how nutrients influence immune cell function and inflammatory networks, and discuss ways to integrate evidence-based nutritional strategies into contemporary pulmonary rehabilitation frameworks. Specifically, we will examine how these nutrients may serve as adjuncts to conventional rehabilitation, not merely by suppressing inflammation, but by addressing specific functional deficits such as respiratory muscle weakness, exercise intolerance, and impaired quality of life that are common in children recovering from severe or chronic lung disease. To graphically summarize this integrative perspective, Figure 1 provides a schematic overview of how these key nutrients modulate the gut-lung axis and immune networks to ultimately improve outcomes in pediatric pulmonary rehabilitation. Ultimately, this synthesis seeks to provide a theoretical foundation and practical guidance for developing individualized and comprehensive therapeutic approaches for children with chronic lung diseases.

## 2 The role of key nutrients in immunomodulation for pediatric pulmonary health

### 2.1 Vitamin D

Vitamin D, a crucial fat-soluble vitamin, exerts profound influence over both innate and adaptive immunity. Its active metabolite, 1,25-dihydroxyvitamin D, binds to and activates vitamin D receptors distributed throughout the body, acting as a central regulator of immune responses (8).

Within the innate immune system, vitamin D enhances the phagocytic capacity of monocytes and macrophages while stimulating the production of antimicrobial peptides such as cathelicidin and defensins. This dual action strengthens the physical barrier defense of respiratory mucosa. Concurrently, vitamin D helps restrain excessive release of pro-inflammatory cytokines, including TNF-α and IL-6, thereby mitigating inflammation-induced tissue damage (9–11).

In adaptive immunity, vitamin D modulates dendritic cell differentiation and function, consequently shaping T-cell responses. It suppresses the differentiation of pro-inflammatory Th1 and Th17 cell lineages while promoting the generation of immunosuppressive regulatory T cells (Tregs). This coordinated regulation helps reestablish immune equilibrium and maintain tolerance (12, 13).

Clinical evidence consistently demonstrates that vitamin D deficiency correlates with increased inflammatory burden and functional impairment across various chronic respiratory conditions. In adults with chronic obstructive pulmonary disease (COPD), low vitamin D levels show significant association with higher exacerbation rates, accelerated lung function decline, and elevated systemic inflammatory markers(14). **In pediatric populations**, observational studies indicate that vitamin D status inversely correlates with airway hyperresponsiveness and asthma control in children (15), and a double-blind placebo-controlled trial in asthmatic children demonstrated that vitamin D supplementation may reduce airway reactivity (16).

However, interventional trials with vitamin D supplementation have yielded conflicting outcomes. The large-scale VITAL-Lung trial, conducted in adults, found that long-term vitamin D supplementation failed to significantly reduce COPD exacerbation frequency or slow lung function decline (14). Conversely, other investigations suggest potential benefits for respiratory muscle function and overall ventilatory efficiency. For instance, a study in healthy middle-aged adults reported that combined low-dose vitamin D and ω-3 fatty acid supplementation in healthy middle-aged adults led to modest improvements in pulmonary function parameters, including vital capacity (17). These discrepant findings likely reflect differences in baseline vitamin D status, patient populations, and intervention protocols.

Preclinical studies provide compelling mechanistic support for vitamin D's role in pulmonary immunity. Vitamin D-deficient mouse models develop more severe lung tissue damage following pathogen exposure, exhibiting elevated inflammatory cytokines (IL-6, TNF-α) and worse respiratory mechanics. Vitamin D supplementation, in turn, appears to regulate antimicrobial peptide synthesis at the genetic level while reducing alveolar protein leakage and neutrophil infiltration (18, 19).

Children—particularly those born preterm, undernourished, or with malabsorptive conditions—represent a high-risk population for vitamin D deficiency (20). Recent research has begun exploring vitamin D's role in neonatal acute respiratory distress syndrome and its interactions with the gut-lung axis. Although observational studies during the COVID-19 pandemic suggested links between low vitamin D status and severe disease outcomes, well-designed randomized controlled trials specifically in pediatric populations remain scarce (18). From a rehabilitation perspective, the potential of vitamin D to enhance respiratory muscle contractility and reduce exacerbation frequency is particularly relevant; even modest reductions in respiratory infections can prevent interruptions in physical therapy and preserve functional gains achieved during rehabilitation programs (21).

Moving forward, prospective studies must enroll children across different ages and disease stages to establish optimal timing, dosage, and duration of vitamin D supplementation. Future research should also account for baseline nutritional status and genetic backgrounds to enable truly personalized therapeutic approaches.

### 2.2 Omega-3 fatty acids

Omega-3 polyunsaturated fatty acids (PUFAs), particularly eicosapentaenoic acid (EPA) and docosahexaenoic acid (DHA), represent essential immunomodulatory nutrients with far-reaching implications for pulmonary health. These fatty acids incorporate into cell membrane phospholipids, modifying membrane fluidity and serving as precursors for specialized pro-resolving mediators (SPMs)—key players in initiating, maintaining, and ultimately resolving inflammatory responses.

The fundamental mechanism of omega-3 PUFAs lies in their potent anti-inflammatory and pro-resolving activities. Their metabolic derivatives, including Resolvin D1 and Protectin D1, effectively restrain excessive neutrophil infiltration, promote the transition of macrophages from pro-inflammatory to repair phenotypes, and enhance clearance of apoptotic cells and pathogens. Through these coordinated actions, they actively drive the resolution of inflammation and facilitate tissue repair (22–24).

At the molecular level, EPA and DHA compete with omega-6 PUFAs (such as arachidonic acid) for metabolic enzymes, thereby reducing the production of pro-inflammatory eicosanoids. Additionally, they suppress the secretion of pro-inflammatory cytokines like IL-6 and CXCL8 in response to viral or bacterial stimulation, partly through modulating p38 MAPK signaling pathways (25).

Clinical investigations reveal that omega-3 supplementation demonstrates potential for improving inflammatory profiles and functional outcomes across various pulmonary conditions, though the evidence base is heavily weighted toward adult populations, with pediatric data remaining sparse. In adults with COPD undergoing rehabilitation, combining omega-3 fatty acids with leucine and vitamin D alongside high-intensity exercise training appears to yield synergistic benefits, particularly in enhancing inspiratory muscle strength, lean body mass, and exercise tolerance (26). However, in individuals with cystic fibrosis—a condition spanning both pediatric and adult populations—a randomized, multicenter, double-blind, placebo-controlled trial found that long-term DHA supplementation failed to demonstrate significant improvements in FEV1 or other crucial inflammatory indices(27), a finding corroborated by systematic reviews (28, 29).

Encouragingly, pediatric-specific evidence is beginning to emerge. In a cross-sectional study of obese minority adolescents with asthma, higher serum levels of n-3 PUFAs correlated with better lung function parameters and reduced insulin resistance, suggesting that omega-3 fatty acids may protect airway function through both metabolic and immunologic pathways in this specific pediatric subgroup (30).

Preclinical studies further substantiate these protective effects. Animal models demonstrate that omega-3 supplementation alleviates bleomycin-induced pulmonary fibrosis, reduces lung inflammation and injury following environmental cadmium exposure or pathogen challenge, and improves outcomes in various other lung injury models. Particularly promising are nanoemulsion formulations, which show enhanced efficacy due to improved bioavailability (31–33).

Several practical considerations warrant emphasis. The source, dosage, and formulation of omega-3 supplements critically influence their effectiveness. While algal oils and fish oils provide direct sources of EPA and DHA, plant-derived alpha-linolenic acid undergoes limited conversion to these active forms in humans. Advanced delivery systems, such as those utilizing nanotechnology, may enhance lung tissue deposition and bioavailability, potentially yielding superior anti-inflammatory and anti-fibrotic outcomes (34).

Consequently, identifying optimal sources, formulations, and dosing regimens for omega-3 supplementation represents an important direction for future clinical research and application in pediatric pulmonary rehabilitation.

### 2.3 Prebiotics/Probiotics

As key modulators of microbial ecology, prebiotics and probiotics demonstrate unique value in pediatric pulmonary immunoregulation through the bidirectional communication network known as the gut-lung axis.

Accumulating evidence reveals that the gut and lungs are not isolated organs, but rather interconnected through circulatory, lymphatic, and shared mucosal immune pathways, forming a dynamic gut-lung axis (35, 36). When gut microbiota ferment dietary fibers or human milk oligosaccharides, the resulting short-chain fatty acids (SCFAs) enter systemic circulation via the portal vein, enhancing alveolar macrophage bactericidal activity while simultaneously suppressing TLR-NF-κB signaling. This dual action reduces pulmonary hyperreactivity to pathogen-associated molecular patterns such as lipopolysaccharide (37, 38). Conversely, in severe conditions like ARDS or COVID-19, inflammatory mediators and oxidative stress signals released in the lungs can travel via vagal nerve and circulatory pathways to disrupt intestinal tight junctions and reduce beneficial bacterial colonization (e.g., Bifidobacterium), establishing a vicious cycle of “lung-injuring-gut, gut-then-further-harming-lung” (39, 40). Consequently, microbial dysbiosis transcends gastrointestinal concerns to become a critical “rate-limiting step” determining whether preterm infants develop bronchopulmonary dysplasia or critically ill patients successfully wean from mechanical ventilation.

Preclinical studies consistently demonstrate that administering prebiotics (e.g., fructooligosaccharides, galactooligosaccharides) or probiotics containing Lactobacillus and Bifidobacterium strains significantly increases fecal butyrate concentrations within 7–14 days, restores gut barrier integrity, and reduces circulating IL-6 and TNF-α levels. Notably, this anti-inflammatory effect parallels that of low-dose corticosteroids while avoiding systemic immunosuppression (41, 42).

Clinical evidence further bridges mechanism and outcome. A comprehensive meta-analysis by Williams et al., encompassing 58 randomized controlled trials and nearly 12,000 infants, found that prebiotic/synbiotic interventions reduced the odds ratio for ≥1 respiratory tract infection to 0.73 while enhancing natural killer cell cytotoxicity by 0.74 standard deviations. Although a small double-blind trial in HIV-infected children showed that 4-week synbiotic supplementation combined with ω-3/6 fatty acids and amino acids failed to significantly reduce hs-CRP or CD38+HLA-DR+ T cells, the strong covariation between key genera (Clostridia, Bacteroides) and inflammatory markers suggests substantial potential for precisely targeted microbial interventions. Observational studies in severe COVID-19 patients indicate that complex probiotics containing Bifidobacterium lactis and Lactobacillus plantarum, when combined with EPA, vitamin D, and whey protein, reduce intestinal permeability biomarker (I-FABP) by 40% and shorten hospital stays by an average of 4.3 days. While awaiting large-scale RCTs, these findings outline a promising clinical prospect for “treating the lung via the gut.”

Collectively, prebiotics and probiotics employ multiple mechanisms to support pulmonary recovery: upregulating mucosal protective factors (TGF-β, IL-10), rebalancing Th1/Th2 responses, inhibiting the NLRP3 inflammasome via the SCFA-GPR43 axis, enhancing macrophage phagocytosis and NK cell cytotoxicity, and expanding the Treg pool. Together, these actions establish a safe, sustainable, and early-initiable “microecological nutritional immunology” strategy for pulmonary rehabilitation.

### 2.4 Amino acids and proteins

Amino acids and proteins play fundamental roles in pediatric pulmonary rehabilitation by providing essential building blocks for immune molecules and maintaining respiratory muscle function. Branched-chain amino acids (BCAAs)—leucine, isoleucine, and valine—serve as crucial activators of muscle protein synthesis via the mTOR signaling pathway, effectively countering disease-associated muscle atrophy. Their metabolite, β-hydroxy-β-methylbutyrate (HMB), further demonstrates the ability to reduce muscle protein breakdown and enhance regenerative capacity under inflammatory conditions by modulating NF-κB and other signaling pathways (43, 44). From a pulmonary rehabilitation perspective, the role of amino acids extends beyond systemic immune support to direct functional enhancement. The diaphragm and intercostal muscles are skeletal muscles susceptible to disuse atrophy and catabolic wasting during severe respiratory illness or prolonged mechanical ventilation. Supplementation with branched-chain amino acids, particularly leucine, provides a targeted metabolic intervention to support respiratory muscle protein synthesis and mitochondrial biogenesis. This has direct implications for rehabilitation outcomes: improved respiratory muscle strength translates to enhanced cough efficacy, reduced atelectasis, more effective airway clearance, and better tolerance of physical therapy sessions. Therefore, combining amino acid supplementation with exercise-based pulmonary rehabilitation exemplifies a “nutrient-enhanced physiotherapy” approach, wherein nutritional support directly amplifies the functional gains achievable through training (45, 46).

Clinical evidence from adult populations provides a rationale for investigating similar strategies in children. In geriatric patients with sarcopenia, BCAA supplementation combined with vitamin D and low-intensity resistance training significantly improves muscle strength, functional independence, and nutritional status (47), suggesting potential value for similar strategies in pulmonary rehabilitation where respiratory muscle recovery is crucial. Additionally, a recent study in critically ill postoperative children demonstrated that early enteral protein supplementation significantly improved nitrogen balance without adverse effects (48), providing pediatric-specific proof-of-concept for protein-based nutritional support in the acute care setting.

Regarding inflammation regulation, amino acid supplementation exhibits multifaceted immunomodulatory properties. In a Mycobacterium tuberculosis-infected mouse model, either ω-3 fatty acids or iron supplementation alone reduced pulmonary and systemic levels of pro-inflammatory cytokines (IL-1β, TNF-α, IL-6), but their combination paradoxically attenuated these anti-inflammatory effects, highlighting the complexity of nutrient interactions and the need for careful formulation (49). In COPD patients, EPA/DHA supplementation induces favorable changes in whole-body amino acid metabolism, manifested as reduced protein catabolism and increased systemic production of certain conditionally essential amino acids (50**?** , 51).

In clinical practice, amino acid and protein supplementation should be integrated throughout the rehabilitation process: early initiation of high-protein enteral or parenteral nutrition enriched with BCAAs for postoperative or nutritionally at-risk children (52)—a strategy supported by Yoga Devaera et al.'s (48) report demonstrating significantly improved nitrogen balance without adverse effects in critically ill postoperative children; and targeted supplementation with leucine or its metabolite HMB during exercise rehabilitation to specifically enhance protein synthesis and functional recovery in respiratory and other skeletal muscles (53).

Therefore, combining amino acids with anti-inflammatory nutrients like ω-3 fatty acids may create synergistic effects through coordinated regulation of protein metabolism and inflammatory responses, achieving dual enhancement of “immunonutrition” and “muscle nutrition” to establish a solid metabolic and functional foundation for pediatric pulmonary rehabilitation. The immunomodulatory mechanisms of the key nutrients discussed in this section (Vitamin D, Omega-3 fatty acids, Pre/Probiotics, and Amino Acids) are summarized in Table 1, which provides a consolidated overview of their primary targets and molecular effects within the context of pediatric pulmonary immunity.

**Table 1:** Core molecular mechanisms in pediatric vs. adult TBI repair.

Dimension	Pediatric mechanisms	Adult mechanisms	Representative downstream effects	Key evidence (Ref #)
Repair strategy	Developmental plasticity-driven	Network stability-focused	Pediatric: Promotes neural circuit reorganization. Adult: Limits rewiring to preserve existing networks.	(13–16) vs [17,22–23,79]
BDNF expression	↑ (Age–dependent serum elevation)	↓ (Promoter hypermethylation)	Pediatric: Supports synaptic growth & survival. Adult: Contributes to synaptic dysfunction & cognitive deficits.	[70–71] vs [79,81]
IGF−1 regulation	↑ (Microglial–derived pro–neurogenesis)	↓ (miR−129–mediated suppression)	Pediatric: Accelerates synaptic formation. Adult: Restricts regenerative capacity, predisposing to pyroptosis.	(17, 21) vs (46, 47)
Key miRNAs	miR−124↑ (Neurogenic), miR−133b↑ (Axon regeneration)	miR−129↑ (Pro–pyroptotic), miR−21↑ (Gliosis control)	Pediatric: miR−124 drives neurogenesis; miR−133b aids repair. Adult: miR−129 inhibits IGF−1; miR−21 modulates scar.	[41–42, 75] vs (37–40, 50**?** )
Epigenetic control	DNA hypomethylation (High flexibility)	BDNF/neurotrophin hypermethylation	Pediatric: Permits broad gene expression for plasticity. Adult: Silences pro–regenerative genes.	[78] vs [81]
Pathological outcome†	Abnormal circuitry risk (~40–60% acute ventriculomegaly)	Glial scar dominance (Significant scar formation in >80% chronic TBI)	Pediatric: Maladaptive wiring. Adult: Physical barrier to regeneration.	Clinical & histological studies (3, 8, 57) †

**↑**indicates upregulation, increased expression, or enhanced activity/flexibility relative to the comparison group.

↓indicates downregulation, decreased expression, or reduced activity/flexibility relative to the comparison group.

†Pathological outcome notes: The data for children were obtained from a clinical imaging study (57), which reported a ventricular enlargement rate of 40–60%. The data for adults were derived from histological analyses [39, 80].

## 3 Impact of nutritional interventions on reinfection risk and immunological memory

Beyond modulating acute-phase immune responses, nutritional status plays a fundamental role in shaping long-term immunological memory and reducing susceptibility to recurrent infections. Specific nutrients provide the essential building blocks for establishing durable and efficient adaptive immunity, either through direct regulation of immune cell differentiation and function or indirectly by optimizing the microecological environment.

At the level of immunological memory formation, both vitamin D and ω-3 fatty acids demonstrate significant regulatory effects on the survival and functional maintenance of memory T and B cells. Vitamin D promotes the generation of regulatory T cells and influences T-cell differentiation pathways, thereby potentially enhancing the quality of immunological memory (54, 55). While primarily recognized for their anti-inflammatory and pro-resolving properties, ω-3 fatty acids—through metabolites such as Resolvins D1/D2 acting via ALX/FPR2 receptors—also suppress the expansion of pro-inflammatory Th1/Th17 memory T cells while promoting Foxp3^+^ Treg differentiation and long-term survival. This dual activity redirects immune memory toward an anti-inflammatory, tolerance-favoring phenotype, achieving simultaneous inflammation resolution and memory quality optimization (56–60). Notably, complex lipid mixtures derived from whole-food sources, such as enzymatically modified fish oils rich in multiple long-chain fatty acids, exhibit broad regulatory effects on interferon signaling and cytokine networks in animal models. These findings suggest that such natural complexes may offer unique advantages in establishing more robust and resilient immunological memory patterns (34, 61).

The translational potential of these mechanistic insights is supported by a growing, though heterogeneous, body of clinical evidence. Selected key studies highlighting the effects of nutritional interventions on respiratory outcomes and immunity are summarized in Table 2. Clinical evidence supporting the role of nutrition in reducing reinfection risk is particularly compelling in pediatric respiratory infections, though it should be noted that much of the supporting mechanistic data derives from adult or preclinical studies. A large systematic review and meta-analysis demonstrated that prebiotic or synbiotic supplementation significantly decreases the incidence of recurrent respiratory tract infections while enhancing natural killer cell activity; importantly, this analysis included nine pediatric-specific synbiotic studies, providing direct evidence in children (62). This finding is corroborated by a randomized controlled trial in preterm infants by Luoto and colleagues, where early prebiotic/probiotic intervention substantially reduced the risk of recurrent viral respiratory infections (63).

**Table 2:** Age–adapted precision intervention strategies for TBI repair. Con.

Strategy type	Pediatric applications	Adult applications	Mechanism	Evidence level & Key support
Molecular delivery	Exosomal miR−133b	CRISPR/dCas9–BDNF editing	Neuronal differentiation/Epigenetic regulation	Preclinical [87]
Physical stimulation	Electroacupuncture	Electroacupuncture	BDNF–miRNA axis modulation	Preclinical [88,89]
Growth factors	IGF−1 enhancement^*^	Not Recommended †	Neurogenesis promotion	Preclinical (Hypoxia model) [72]
Natural compounds	Ganglioside GM1	Curcumin	Anti–inflammatory & BDNF modulation	Preclinical [92]
Cross–system modulators	Probiotics (*Bifidobacterium*)	SCFA supplementation	Gut–brain axis metabolic reprogramming	Hypothesized (62, 65) ‡

Evidence Limitations:

^*^ The evidence for IGF−1 is derived exclusively from the neonatal hypoxia model [73], and the extrapolation of these findings to TBI in children requires further validation.

^†^ Not Recommended for Adults: Direct IGF−1 enhancement is not advised due to a lack of supportive evidence in adult TBI, potential suppression by miRNAs like miR−129, and age–associated risks such as increased tumor promotion concerns.

‡ Hypothesized Mechanism: The gut–brain axis role is a compelling hypothesis derived from correlative and preliminary experimental data. Its precise mechanistic role in TBI repair and efficacy as a therapeutic target require further validation in age–stratified animal models and clinical studies.

The balance of dietary fatty acids emerges as another critical factor. Observational studies indicate that increased intake of plant-based ω-3 alpha-linolenic acid (ALA), particularly in the context of low ω-6 fatty acid consumption, correlates with reduced chronic respiratory symptoms such as coughing and wheezing (64). This suggests that the ω-6/ω-3 ratio may be a key determinant of airway immune homeostasis and susceptibility to reinfection.

During the COVID-19 pandemic, integrated nutritional support strategies—typically combining vitamin D, zinc, ω-3 fatty acids, and arginine—showed promise in preliminary clinical observations for shortening viral clearance time and reducing disease progression risk. These benefits appear partly attributable to the nutrients' synergistic stabilization of immune homeostasis and enhanced pathogen clearance capacity (6, 65, 66). However, these observations require confirmation through rigorously designed, large-scale intervention studies specifically in pediatric populations.

In summary, strategically optimizing immunological memory through scientifically grounded nutritional interventions represents a crucial approach for reducing reinfection risk in children with pulmonary diseases and achieving sustainable rehabilitation benefits.

## 4 Challenges in clinical translation and future research directions

Despite the considerable promise of immunonutrition in pediatric pulmonary rehabilitation, translating existing evidence into clinical practice faces several substantial challenges. Most current research focuses on adult or elderly populations, while pediatric-specific studies remain limited in number and typically involve small-sample, single-center trials that restrict generalizability. Significant heterogeneity in intervention protocols—including variations in nutrient composition, dosage, and treatment duration—combined with inadequate systematic assessment of baseline nutritional status or monitoring of objective biomarkers such as plasma nutrient levels, complicates accurate interpretation and cross-study comparison. Furthermore, multiple systematic reviews have highlighted prevalent methodological limitations in this field, including high risk of bias and generally low quality of evidence, underscoring the need for greater scientific rigor.

Given the extreme heterogeneity in study design, dosing protocols, and patient characteristics, it would be premature to propose specific dosing ranges or clinical algorithms at this stage. Rather, the immediate priority is to establish standardized pediatric research frameworks to address these foundational questions. Nevertheless, synthesizing the available evidence does allow for the identification of high-risk pediatric subgroups that should be prioritized in future investigation. These subgroups include: (1) preterm infants, who are at elevated risk for bronchopulmonary dysplasia and vitamin D deficiency; (2) children with cystic fibrosis, who experience chronic inflammation, malabsorption, and demonstrated associations between vitamin D status and pulmonary outcomes; (3) obese adolescents with asthma, in whom altered fatty acid metabolism and systemic inflammation may render immunonutritional interventions particularly impactful; and (4) children with recurrent respiratory tract infections, who may benefit from gut-lung axis modulation via prebiotics and probiotics. These populations represent logical targets for early-phase clinical trials aimed at evaluating the feasibility and preliminary efficacy of immunonutritional adjuncts to pulmonary rehabilitation. It is important to emphasize that this identification of priority subgroups is intended to guide future research agendas, not to imply that evidence-based clinical recommendations currently exist for these populations in the context of pulmonary rehabilitation.

To advance beyond these limitations and bridge the gap between theoretical potential and practical application, future research should prioritize several key directions:

First, establishing standardized assessment systems and precision nutrition frameworks is imperative. This requires developing validated nutritional risk screening tools specifically for pediatric pulmonary rehabilitation populations. Future study designs should routinely incorporate dynamic monitoring of nutritional biomarkers—such as 25(OH)D, Omega-3 Index, and short-chain fatty acids—while stratifying participants according to baseline nutritional status to enable truly personalized interventions.

Second, promoting high-quality combined intervention studies through well-designed, multicenter, large-sample randomized controlled trials is essential. These trials should specifically investigate the synergistic effects of core nutrient combinations—including vitamin D, ω-3 fatty acids, prebiotics/probiotics, and branched-chain amino acids—alongside conventional rehabilitation training. Study endpoints must comprehensively capture multidimensional outcomes spanning pulmonary function, inflammatory markers, muscle function, reinfection rates, and quality of life measures.

Third, innovating delivery technologies and dietary sources represents a crucial frontier. Advanced formulation strategies such as nanotechnology and microencapsulation should be leveraged to enhance the bioavailability and lung-targeting efficiency of lipophilic nutrients like ω-3 fatty acids and vitamin D. Concurrently, systematic evaluation of efficacy differences between plant-based nutrients (such as ALA and DPA) and animal-derived alternatives will help expand safe and sustainable nutritional sources.

Finally, integrating multi-omics and digital health technologies offers transformative potential. Future work should explore gut-lung axis and systemic metabolomic biomarkers to identify intervention targets and assess the comprehensive effects of nutritional strategies. Combining wearable devices with digital health platforms will enable dynamic, objective monitoring of long-term adherence, safety, and efficacy responses in pediatric patients.

While direct evidence in pediatric pulmonary rehabilitation is lacking, existing data allow the formulation of a testable conceptual framework for how immunonutritional support might eventually be phased in parallel with rehabilitation progression. As a hypothesis for future investigation rather than a clinical guideline, this framework proposes: (1) early recovery—anti-inflammatory support via vitamin D and omega-3 fatty acids to stabilize clinical status; (2) active training—anabolic support via branched-chain amino acids to enhance exercise tolerance and respiratory muscle gains; and (3) long-term maintenance—dietary optimization to sustain immunological resilience. In clinical practice, a stepwise approach aligned with this conceptual model may be considered, beginning with comprehensive nutritional assessment for all children entering pulmonary rehabilitation. For those identified as nutritionally at-risk—particularly within the high-risk subgroups described above—targeted supplementation may be warranted, with doses guided by available pediatric evidence where it exists and by cautious extrapolation from adult studies where it does not. However, given the limitations of the current evidence base, any such supplementation should be regarded as provisional, applied with appropriate clinical judgment, and accompanied by regular monitoring of both serum biomarkers and functional outcomes. This proposed model and its clinical translation require validation through prospective pediatric trials before definitive adoption. Ultimately, successful translation will depend on strengthening multidisciplinary collaboration. Bringing together the complementary expertise of nutritionists, respiratory therapists, pediatricians, and microbiome specialists will be essential for developing and executing truly individualized, precision immunonutrition strategies. Only through such integrated approaches can we fully realize the dual objectives of nutritional support and immune optimization in pediatric pulmonary rehabilitation. To address the current challenges and systematically advance the field, we have synthesized the primary research priorities and practical implementation strategies into a structured framework (Table 3).

**Table 3:** Critical research gaps and future directions in age–stratified TBI repair.

Challenge domain	Current limitations	Proposed solutions	Expected outcomes
Pediatric data scarcity	Limited access to brain tissue/CSF samples (ethical constraints)	Multicenter collaboration: Organoid models + Non–invasive biomarker development	Pediatric TBI molecular atlas
Cross–system mechanisms	Unclear interactions between gut–brain axis/CSF dynamics and BDNF networks	Humanized murine models + Gnotobiotic animal studies	Age–dependent weight of systemic modulation
Intervention translation	Low miRNA delivery efficiency (< 5% CNS penetration due to BBB)	Engineered nanocarriers (exosome–liposome hybrids)	Enhanced CNS–targeted delivery specificity
Age–stratified efficacy	Lack of clinical trial designs adapted to developmental stages	Staggered dosing trials (pediatric–first → adult expansion)	Precision therapeutic windows

BBB, Blood–brain barrier; CSF, Cerebrospinal fluid; CNS, Central nervous system.

Evidence Support:

All content derived from Section 5: Limitations and Future Perspectives (Original analysis).

Key quantitative claims (e.g., miRNA delivery < 5%) sourced to [102-103].

## 5 Conclusion

Nutritional immunomodulation serves as an indispensable adjunct in pediatric pulmonary rehabilitation, with its importance increasingly recognized through both basic research and clinical observation. By targeting specific rehabilitation endpoints—respiratory muscle function, exercise capacity, infection-related interruptions, and quality of life—immunonutrition offers a complementary pathway to enhance outcomes beyond what can be achieved through physical and respiratory therapy alone. This review has systematically examined how key nutrients—particularly vitamin D, ω-3 fatty acids, prebiotics/probiotics, and branched-chain amino acids—contribute to lung tissue repair, respiratory muscle function, and reduced reinfection risk. These benefits appear mediated through multiple interconnected mechanisms: modulating immune cell function, optimizing inflammatory responses, and enhancing communication along the gut-lung axis.

Nevertheless, significant limitations persist within the current evidence base. High-quality, large-scale clinical studies specifically designed for pediatric populations remain scarce, while substantial heterogeneity across intervention protocols complicates the development of standardized clinical guidelines. These knowledge gaps highlight the need for more rigorous and targeted research in this emerging field.

The future advancement of this discipline hinges on transitioning from single-nutrient investigations toward multidimensional, personalized nutritional strategies. Well-designed multicenter randomized controlled trials are needed to verify the synergistic potential of combined nutrient interventions. Integrating nutritional biomarker monitoring, microbial ecology analysis, and multi-omics technologies will be essential for establishing precision nutrition pathways in clinical practice. Meanwhile, novel delivery systems and digital health technologies offer promising tools for enhancing both intervention efficacy and long-term adherence.

In clinical settings, we recommend incorporating comprehensive nutritional assessment as a standard component of all pediatric pulmonary rehabilitation programs. Evidence-based, stage-appropriate nutritional support should then be implemented according to the best available data. Crucially, achieving optimal outcomes will require close collaboration among multidisciplinary teams including pediatricians, respiratory therapists, nutritionists, and microbiome specialists.

Through such integrated approaches, we can effectively translate theoretical advances in immunonutrition into tangible clinical benefits. By simultaneously optimizing immune function and nutritional status, we may fundamentally improve long-term quality of life for children living with chronic pulmonary conditions.

## References

[1] Verduci E Köglmeier J. Immunomodulation in children: the role of the diet. J Pediatr Gastroenterol Nutr. (2021) 73:293–8. doi: 10.1097/MPG.0000000000003152 33872290 PMC9770123

[2] Verduci E D'Auria E Bosetti A Vizzuso S Milanta C Pendezza E . Immunomodulatory diet in pediatric age. Minerva Pediatr. (2021) 73:128–49. doi: 10.23736/S2724-5276.21.06214-9 33880904

[3] Piersigilli F Van Grambezen B Hocq C Danhaive O. Nutrients and Microbiota in Lung Diseases of Prematurity: The Placenta-Gut-Lung Triangle. Nutrients. (2020) 12:469. doi: 10.3390/nu12020469 32069822 PMC7071142

[4] Di Renzo L Gualtieri P Pivari F Soldati L Attinà A Leggeri C . COVID-19: Is there a role for immunonutrition in obese patient? J Transl Med. (2020) 18:415. doi: 10.1186/s12967-020-02594-4 33160363 PMC7647877

[5] Pecora F Persico A Argentiero A Neglia C Esposito S. The Role of Micronutrients in Support of the Immune Response against Viral Infections. Nutrients. (2020) 12:3198. doi: 10.3390/nu12103198 33092041 PMC7589163

[6] Diyya ASM Thomas NV. Multiple Micronutrient Supplementation: As a Supportive Therapy in the Treatment of COVID-19. Biomed Res Int. (2022) 2022:3323825. doi: 10.1155/2022/3323825 35355818 PMC8960013

[7] P Calder PC Carr AC Gombart AF Eggersdorfer M. Optimal Nutritional Status for a Well-Functioning Immune System Is an Important Factor to Protect against Viral Infections. Nutrients. (2020) 12:1181. doi: 10.3390/nu12041181

[8] Hamza FN Daher S Fakhoury HMA Grant WB Kvietys PR Al-Kattan K. Immunomodulatory Properties of Vitamin D in the Intestinal and Respiratory Systems. Nutrients. (2023) 15:1696. doi: 10.3390/nu15071696 37049536 PMC10097244

[9] Small AG Harvey S Kaur J Putty T Quach A Munawara U . Vitamin D upregulates the macrophage complement receptor immunoglobulin in innate immunity to microbial pathogens. Commun Biol. (2021) 4:401. doi: 10.1038/s42003-021-01943-3 33767430 PMC7994403

[10] Gombart AF. The vitamin D-antimicrobial peptide pathway and its role in protection against infection. Future Microbiol. (2009) 4:1151–65. doi: 10.2217/fmb.09.87 19895218 PMC2821804

[11] Gao D Trayhurn P Bing C. 1,25-Dihydroxyvitamin D3 inhibits the cytokine-induced secretion of MCP-1 and reduces monocyte recruitment by human preadipocytes. Int J Obes. (2013) 37:357–65. doi: 10.1038/ijo.2012.53 PMC3428854 22508334

[12] Fisher SA Rahimzadeh M Brierley C Gration B Doree C Kimber CE . The role of vitamin D in increasing circulating T regulatory cell numbers and modulating T regulatory cell phenotypes in patients with inflammatory disease or in healthy volunteers: A systematic review. PLoS One. (2019) 14:e0222313. doi: 10.1371/journal.pone.0222313 31550254 PMC6759203

[13] Li B Zhang X Sun Z Xu B Wu J Liu H . A Novel Strategy for the Treatment of Allergic Rhinitis: Regulating Treg/Th17 and Th1/Th2 Balance *In Vivo* by Vitamin D. Comput Math Methods Med. (2022) 2022:9249627. doi: 10.1155/2022/9249627 35959353 PMC9357782

[14] Gold DR Carey VJ Hersh CP Wan E Camargo CA Jr Lee IM . Vitamin D Supplementation, Chronic Obstructive Lung Disease and Asthma Exacerbations, and Lung Function Decline. J Nutr. (2025) 155:1417–28. doi: 10.1016/j.tjnut.2025.02.003 39922497 PMC12121404

[15] Hatami G Ghasemi K Motamed N Firoozbakht S Movahed A Farrokhi S. Relationship between Vitamin D and Childhood Asthma: A Case-Control Study. Iran J Pediatr. (2014) 24:710–4. 26019776 PMC4442832

[16] Bar Yoseph R Livnat G Schnapp Z Hakim F Dabbah H Goldbart A . The effect of vitamin D on airway reactivity and inflammation in asthmatic children: A double-blind placebo-controlled trial. Pediatr Pulmonol. (2015) 50:747–53. doi: 10.1002/ppul.23076 24989842

[17] Sujeta A Capkauskiene S Vizbaraite D Stasiule L Balciunas M Stasiulis A . Low-Dose Omega-3 Fatty Acid and Vitamin D for Anthropometric, Biochemical Blood Indices and Respiratory Function. Does it work? Int J Vitam Nutr Res. (2020) 90:67-83. doi: 10.1024/0300-9831/a000476 30932776

[18] Mardi A Kamran A Pourfarzi F Zare M Hajipour A Doaei S . Potential of macronutrients and probiotics to boost immunity in patients with SARS-COV-2: a narrative review. Front Nutr. (2023) 10:1161894. doi: 10.3389/fnut.2023.1161894 37312883 PMC10259402

[19] Shi YY Liu TJ Fu JH Xu W Wu LL Hou AN . Vitamin D/VDR signaling attenuates lipopolysaccharide-induced acute lung injury by maintaining the integrity of the pulmonary epithelial barrier. Mol Med Rep. (2016) 13:1186–94. doi: 10.3892/mmr.2015.4685 26675943 PMC4732862

[20] Guo H Xie J Yu X Tian Y Guan M Wei J. Effects of vitamin D supplementation on serum 25(OH)D(3) levels and neurobehavioral development in premature infants after birth. Sci Rep. (2024) 14:23972. doi: 10.1038/s41598-024-75191-w 39397102 PMC11471846

[21] Rafiq R Prins HJ Boersma WG Daniels JM den Heijer M Lips P . Effects of daily vitamin D supplementation on respiratory muscle strength and physical performance in vitamin D-deficient COPD patients: a pilot trial. Int J Chron Obstruct Pulmon Dis. (2017) 12:2583–92. doi: 10.2147/COPD.S132117 PMC5584776 28894361

[22] Bodur M Yilmaz B Agagündüz D Ozogul Y. Immunomodulatory Effects of Omega-3 Fatty Acids: Mechanistic Insights and Health Implications. Mol Nutr Food Res. (2025) 69:e202400752. doi: 10.1002/mnfr.202400752 40159804 PMC12087734

[23] Wierenga KA Strakovsky RS Benninghoff AD Rajasinghe LD Lock AL Harkema JR . Requisite Omega-3 HUFA Biomarker Thresholds for Preventing Murine Lupus Flaring. Front Immunol. (2020) 11:1796. doi: 10.3389/fimmu.2020.01796 32973753 PMC7473030

[24] Dominguez EC Heires AJ Pavlik J Larsen TD Guardado S Sisson JH . A High Docosahexaenoic Acid Diet Alters the Lung Inflammatory Response to Acute Dust Exposure. Nutrients. (2020) 12:2334. doi: 10.3390/nu12082334 32759853 PMC7468878

[25] Rutting S Zakarya R Bozier J Xenaki D Horvat JC Wood LG . Dietary Fatty Acids Amplify Inflammatory Responses to Infection through p38 MAPK Signaling. Am J Respir Cell Mol Biol. (2019) 60:554-568. doi: 10.1165/rcmb.2018-0215OC 30648905

[26] van de Bool C Rutten EPA van Helvoort A Franssen FME Wouters EFM Schols AMWJ. A randomized clinical trial investigating the efficacy of targeted nutrition as adjunct to exercise training in COPD. J Cachexia Sarcopenia Muscle. (2017) 8:748-758. doi: 10.1002/jcsm.12219 PMC5659064 28608438

[27] López-Neyra A Suárez L Muñoz M de Blas A Ruiz de Valbuena M Garriga M . Long-term docosahexaenoic acid (DHA) supplementation in cystic fibrosis patients: a randomized, multi-center, double-blind, placebo-controlled trial. Prostaglandins Leukot Essent Fatty Acids. (2020) 162:102186. doi: 10.1016/j.plefa.2020.102186 33038833

[28] Watson H Stackhouse C. Omega-3 fatty acid supplementation for cystic fibrosis. The Cochrane database of systematic reviews. (2020) 4:Cd002201. doi: 10.1002/14651858.CD002201.pub6 32275788 PMC7147930

[29] Simon MISDS Dalle Molle R Silva FM Rodrigues TW Feldmann M Forte GC. Antioxidant Micronutrients and Essential Fatty Acids Supplementation on Cystic Fibrosis Outcomes: A Systematic Review. J Acad Nutr Diet. (2020) 120:1016-1033.e1. doi: 10.1016/j.jand.2020.01.007 32249071

[30] Tobias TAM Wood LG Rastogi D. Carotenoids, fatty acids and disease burden in obese minority adolescents with asthma. Clin Exp Allergy. (2019) 49:838–46. doi: 10.1111/cea.13391 30908741 PMC6546527

[31] Galdino de Souza D Santos DS Simon KS Morais JAV Coelho LC Pacheco TJA . Longo, Fish Oil Nanoemulsion Supplementation Attenuates Bleomycin-Induced Pulmonary Fibrosis BALB/c Mice. Nanomaterials. (2022) 12:1683. doi: 10.3390/nano12101683 35630905 PMC9145453

[32] Zhao H Chan-Li Y Collins SL Zhang Y Hallowell RW Mitzner W . Pulmonary delivery of docosahexaenoic acid mitigates bleomycin-induced pulmonary fibrosis. BMC Pulm Med. (2014) 14:64. doi: 10.1186/1471-2466-14-64 24742272 PMC3998951

[33] He J Zhang C Zhang Y Zhang J Su X He P. W, et al. Polyunsaturated Fatty Acid Concentrations and Risk of Pneumoconiosis: A Two-sample Mendelian Randomization Study. Biomed Environ Sci. (2024) 37:1328–33. doi: 10.3967/bes2024.166 39667970

[34] Currie T Myklebust C Bjerknes B Framroze B. Assessing the Potential of an Enzymatically Liberated Salmon Oil to Support Immune Health Recovery from Acute SARS-CoV-2 Infection via Change in the Expression of Cytokine, Chemokine and Interferon-Related Genes. Int J Mol Sci. (2024) 25:6917. doi: 10.3390/ijms25136917 39000027 PMC11241394

[35] Wang L Cai Y Garssen J Henricks PAJ Folkerts G Braber S. The Bidirectional Gut-Lung Axis in Chronic Obstructive Pulmonary Disease. Am J Respir Crit Care Med. (2023) 207:1145–60. doi: 10.1164/rccm.202206-1066TR 36883945 PMC10161745

[36] Li X Shang S Wu M Song Q Chen D. Gut microbial metabolites in lung cancer development and immunotherapy: Novel insights into gut-lung axis. Cancer Lett. (2024) 598:217096. doi: 10.1016/j.canlet.2024.217096 38969161

[37] O'Riordan KJ Collins MK Moloney GM Knox EG Aburto MR Fülling C . Short chain fatty acids: Microbial metabolites for gut-brain axis signalling. Mol Cell Endocrinol. (2022) 546:111572. doi: 10.1016/j.mce.2022.111572 35066114

[38] Parada VD De lFMK Landskron G González MJ Quera R Dijkstra G . Short Chain Fatty Acids (SCFAs)-Mediated Gut Epithelial and Immune Regulation and Its Relevance for Inflammatory Bowel Diseases. Front Immunol. (2019) 10:277. doi: 10.3389/fimmu.2019.01486 30915065 PMC6421268

[39] Ziaka M Exadaktylos A. Gut-derived immune cells and the gut-lung axis in ARDS. Crit Care. (2024) 28:220. doi: 10.1186/s13054-024-05006-x 38965622 PMC11225303

[40] Ancona G Alagna L Alteri C Palomba E Tonizzo A Pastena A . Gut and airway microbiota dysbiosis and their role in COVID-19 and long-COVID. Front Immunol. (2023)14. 1080043. doi: 10.3389/fimmu.2023.1080043 PMC10030519 36969243

[41] Walton GE van den Heuvel EG Kosters MH Rastall RA Tuohy KM Gibson GR. A randomised crossover study investigating the effects of galacto-oligosaccharides on the faecal microbiota in men and women over 50 years of age. Br J Nutr. (2012) 107:1466–75. doi: 10.1017/S0007114511004697 21910949

[42] Pradana A Sari DK Rusda M Tarigan AP Wiyono WH Soeroso NN . Amin, Probiotics-derived butyric acid may suppress systemic inflammation in a murine model of chronic obstructive pulmonary disease (COPD). Narra J. (2025) 5:e1332. doi: 10.52225/narra.v5i1.1332 40352181 PMC12059839

[43] Engelen MPKJ Simbo SY Ruebush LE Thaden JJ Ten Have GAM Harrykissoon RI . Deutz, Functional and metabolic effects of omega-3 polyunsaturated fatty acid supplementation and the role of beta-hydroxy-beta-methylbutyrate addition in chronic obstructive pulmonary disease: A randomized clinical trial. Clinical nutrition. (2024) 43:2263–2278. doi: 10.1016/j.clnu.2024.08.004 39181037

[44] Takeuchi I Yoshimura Y Shimazu S Jeong S Yamaga M Koga H. Effects of branched-chain amino acids and vitamin D supplementation on physical function, muscle mass and strength, and nutritional status in sarcopenic older adults undergoing hospital-based rehabilitation: A multicenter randomized controlled trial. Geriatr Gerontol Int. (2019) 19:12-17. doi: 10.1111/ggi.13547 30358032

[45] Martínez-Arnau FM Fonfría-Vivas R Buigues C Castillo Y Molina P Hoogland AJ . Effects of Leucine Administration in Sarcopenia: A Randomized and Placebo-controlled Clinical Trial. Nutrients. (2020) 12:932. doi: 10.3390/nu12040932 32230954 PMC7230494

[46] Aldhahir AM Rajeh AMA Aldabayan YS Drammeh S Subbu V Alqahtani JS . Nutritional supplementation during pulmonary rehabilitation in COPD: A systematic review. Chron Respir Dis. (2020) 17:1479973120904953. doi: 10.1177/1479973120904953 PMC7019390 32054293

[47] Cochet C Belloni G Buondonno I Chiara F D'Amelio P. The Role of Nutrition in the Treatment of Sarcopenia in Old Patients: From Restoration of Mitochondrial Activity to Improvement of Muscle Performance, a Systematic Review. Nutrients. (2023) 15:3703. doi: 10.3390/nu15173703 37686735 PMC10490489

[48] Yuniar Lestari KA Pudjiadi AH Alatas FS Devaera Y. Devaera, Effect of postoperative enteral protein supplementation on nitrogen balance in critically ill children. Clin Exp Pediatr. (2025) 68:790–800. doi: 10.3345/cep.2025.00227 40468736 PMC12488283

[49] Nienaber A Baumgartner J Dolman RC Ozturk M Zandberg L Hayford FEA . Omega-3 Fatty Acid and Iron Supplementation Alone, but Not in Combination, Lower Inflammation and Anemia of Infection in Mycobacterium tuberculosis-Infected Mice. Nutrients. (2020) 12:2897. doi: 10.3390/nu12092897 32971969 PMC7551947

[50] Engelen M Jonker R Sulaiman H Fisk HL Calder PC Deutz NEP. ω-3 polyunsaturated fatty acid supplementation improves postabsorptive and prandial protein metabolism in patients with chronic obstructive pulmonary disease: a randomized clinical trial. Am J Clin Nutr. (2022) 116:686–98. doi: 10.1093/ajcn/nqac138 35849009 PMC9437982

[51] Tuna T Samur G. The Role of Nutrition and Nutritional Supplements in the Prevention and Treatment of Malnutrition in Chronic Obstructive Pulmonary Disease: Current Approaches in Nutrition Therapy. Curr Nutr Rep. (2025) 14:21. doi: 10.1007/s13668-025-00613-8 39862339 PMC11762775

[52] Sakuraya M Yamashita K Honda M Niihara M Chuman M Washio M . Early administration of postoperative BCAA-enriched PPN may improve lean body mass loss in gastric cancer patients undergoing gastrectomy. Langenbeck's archives of surgery. (2023) 408:336. doi: 10.1007/s00423-023-03045-6 PMC10457225 37624566

[53] Wilkinson DJ Hossain T Hill DS Phillips BE Crossland H Williams JP . Atherton, Effects of leucine and its metabolite β-hydroxy-β-methylbutyrate on human skeletal muscle protein metabolism. J Physiol. (2013) 591:2911–23. doi: 10.1113/jphysiol.2013.253203 23551944 PMC3690694

[54] Kang SW Kim SH Lee N Lee WW Hwang KA Shin MS . 1,25-Dihyroxyvitamin D3 promotes FOXP3 expression via binding to vitamin D response elements in its conserved noncoding sequence region. J Immunol. (2012) 188:5276-82. doi: 10.4049/jimmunol.1101211 PMC3358577 22529297

[55] Cantorna MT Snyder L Lin YD Yang L. Vitamin D and 1,25(OH)2D regulation of T cells. Nutrients. (2015) 7:3011–21. doi: 10.3390/nu7043011 25912039 PMC4425186

[56] Chiurchiù V Leuti A Dalli J Jacobsson A Battistini L Maccarrone M. Proresolving lipid mediators resolvin D1, resolvin D2, and maresin 1 are critical in modulating T cell responses. Sci Transl Med. (2016) 8:353ra111. doi: 10.1126/scitranslmed.aaf7483 PMC5149396 27559094

[57] Cheng T Ding S Liu S Li X Tang X Sun L. Resolvin D1 Improves the Treg/Th17 Imbalance in Systemic Lupus Erythematosus Through miR-30e-5p. Front Immunol. (2021) 12:668760. doi: 10.3389/fimmu.2021.668760 34093566 PMC8171186

[58] Luan H Wang C Sun J Zhao L Li L Zhou B . Resolvin D1 Protects Against Ischemia/Reperfusion-Induced Acute Kidney Injury by Increasing Treg Percentages via the ALX/FPR2 Pathway. Front Physiol. (2020) 11:285. doi: 10.3389/fphys.2020.00285 32317985 PMC7147344

[59] Chen L Huang Y Chen Y Chen J You X Zou L . Huang, Resolvin D1 promotes the resolution of inflammation in the ACLF rat model by increasing the proportion of Treg cells. Immun Inflamm Dis. (2023) 11:e1076. doi: 10.1002/iid3.1076 38018579 PMC10659757

[60] Bento AF Claudino RF Dutra RC Marcon R Calixto JB. Omega-3 fatty acid-derived mediators 17(R)-hydroxy docosahexaenoic acid, aspirin-triggered resolvin D1 and resolvin D2 prevent experimental colitis in mice. J Immunol. (2011) 1871:957-69. doi: 10.4049/jimmunol.1101305 21724996

[61] Gutiérrez S Svahn SL Johansson ME. Effects of Omega-3 Fatty Acids on Immune Cells. Int J Mol Sci. (2019) 20:5028. doi: 10.3390/ijms20205028 31614433 PMC6834330

[62] Williams LM Stoodley IL Berthon BS Wood LG. The Effects of Prebiotics, Synbiotics, and Short-Chain Fatty Acids on Respiratory Tract Infections and Immune Function: A Systematic Review and Meta-Analysis. Adv Nutr. (2022) 13:167–92. doi: 10.1093/advances/nmab114 34543378 PMC8803493

[63] Luoto R Ruuskanen O Waris M Kalliomäki M Salminen S Isolauri E. Prebiotic and probiotic supplementation prevents rhinovirus infections in preterm infants: a randomized, placebo-controlled trial. J Allergy Clin Immunol. (2014) 133:405–13. doi: 10.1016/j.jaci.2013.08.020 24131826 PMC7112326

[64] Lemoine S CM Brigham EP Woo H Hanson CK McCormack MC Koch A . Omega-3 fatty acid intake and prevalent respiratory symptoms among U.S. adults with COPD. BMC Pulm Med. (2019) 19:97. doi: 10.1186/s12890-019-0852-4 31122230 PMC6533751

[65] Muralidharan J Kashyap S S P Jacob M Ollapally A Idiculla J Raj JM . The effect of l-arginine supplementation on amelioration of oxygen support in severe COVID-19 pneumonia. Clin Nutr ESPEN. (2022) 52:431-435. doi: 10.1016/j.clnesp.2022.09.024 PMC9511895 36513483

[66] Naidu AS Wang CK Rao P Mancini F Clemens RA Wirakartakusumah A . Precision nutrition to reset virus-induced human metabolic reprogramming and dysregulation (HMRD) in long-COVID. NPJ science of food. (2024) 8:19. doi: 10.1038/s41538-024-00267-w 38555403 PMC10981760

